# Flow Diversion for Treatment of Acutely Ruptured Intracranial Aneurysms: A Single Center Experience from 45 Consecutive Cases

**DOI:** 10.1007/s00062-019-00846-5

**Published:** 2019-11-04

**Authors:** Muhammad AlMatter, Marta Aguilar Pérez, Victoria Hellstern, Goran Mitrovic, Oliver Ganslandt, Hansjörg Bäzner, Hans Henkes

**Affiliations:** 1 Neuroradiologische Klinik, Klinikum Stuttgart, Kriegsbergstraße 60, 70174 Stuttgart, Germany; 2 Neurochirurgische Klinik, Klinikum Stuttgart, Stuttgart, Germany; 3 Neurologische Klinik, Klinikum Stuttgart, Stuttgart, Germany; 4 Medizinische Fakultät, der Universität Duisburg-Essen, Essen, Germany

**Keywords:** Subarachnoid hemorrhage, Cerebral aneurysms, Flow diversion, Antiplatelets

## Abstract

**Background:**

Reports about the use of flow diverter stents (FDS) in the acute setting of subarachnoid hemorrhage (SAH) are limited. This article presents a single center experiences based on 45 consecutive cases with emphasis on complication rates and clinical and radiologic outcomes.

**Methods:**

A prospectively maintained database of all cases treated with FDS as a stand-alone or adjunct device was retrospectively reviewed. All patients treated within 30 days of SAH were included. Records were made of clinical presentation, details of endovascular treatment, procedural complications, clinical outcome, and degree of occlusion on follow-up.

**Results:**

In this study 45 patients (48.9% females; mean age 58.8 ± 12.4 years) were included. Flow diversion was performed after a median of 4 days. The procedural complication rate was 13.3% resulting in 2.2% permanent morbidities and 4.4% mortalities. No major hemorrhagic complications related to antiplatelet therapy were recorded. Immediate complete occlusion was achieved in 13.3%. Among survivors, complete occlusion was achieved in 94.6%. Excellent clinical outcome was recorded in 68.9% and 81.6% of the total population and survivors, respectively. There were no records of rebleeding from the target lesions.

**Conclusion:**

Flow diversion is an attractive alternative strategy for management of acutely ruptured aneurysms with high rates of delayed complete occlusion and acceptable complication rates.

## Introduction

Flow diversion greatly expanded the spectrum of treatable intracranial aneurysms by endovascular means [1]. With the appropriate indications and medical management, the use of flow diverter stents (FDS) can result in high rates of complete and permanent aneurysmal occlusion, a feature that has always been a major drawback of traditional endovascular treatment [2–5]. The low porosity and high mesh density of FDS facilitate aneurysmal occlusion by reducing the blood flow into the aneurysms and by serving as a scaffold for endothelialization across the aneurysm neck leading to progressive occlusion and reduced rates of recanalization of the treated aneurysm [6]. The use of FDS in the acute phase for the treatment of acutely ruptured aneurysms has been limited by the need for dual antiplatelet therapy (DAPT) and is usually considered as a last resort option if other modalities are not available or suitable [7]. A recent meta-analysis of 20 studies evaluating the use of FDS for treatment of ruptured aneurysms reported a high rate of durable angiographic occlusion, but the associated complication rate was high [8]. The following report presents experiences with the use of FDS under DAPT in the management of acutely ruptured aneurysms based on 45 consecutive cases treated at a tertiary neurovascular center with a focus on the device-related and DAPT-related complications, occlusion rates, and clinical outcomes.

## Methods

The prospectively maintained institutional database of all cases treated with FDS between mid-2010 and mid-2018 was reviewed to identify patients who were treated in the acute phase of SAH within 30 days from the ictus. Included were all patients who underwent endovascular flow diversion as a stand-alone treatment or as adjunctive to primary coiling. Cases in which flow diversion was performed within 30 days of hemorrhage but in a separate intervention after the initial procedure were also included. Patients who underwent staged flow diversion of the aneurysmal remnant after 30 days were excluded. Records were made of the age at presentation, clinical presentation, morphology and location of the ruptured aneurysm, procedural and general complications, and clinical and radiological outcome according to the latest available follow-up.

### Endovascular Procedure

Catheter angiography for evaluation of the ruptured aneurysm was performed in all cases. The decision on the treatment strategy was made by a consensus between a senior neurointerventionalist and a senior vascular neurosurgeon. All procedures were performed with the patient under general anesthesia using a biplane angiography system (Siemens, Erlangen, Germany). Depending on the degree of vessel elongation and the need for an intermediate catheter, either a 6F or an 8F right femoral approach was used. A bolus dose of heparin was not administered or was postponed until the aneurysm has been partially secured, depending on the length of the procedure. Of the commercially available FDS two were used: the p64 flow modulation device (phenox GmbH, Bochum, Germany) and the Pipeline Embolization Device (PED; Medtronic, Irvine, CA, USA). Depending on the type of the device, either an Excelsior XT-27 (Stryker, Fremont, CA, USA) or a Marksman (Medtronic) microcatheter was used for the delivery of the p64 or the PED, respectively. Multiple projections and rotational angiography were used for assessment of the target aneurysm and the parent vessel and assessment of the appropriate wall apposition of the device after deployment. Calibrated measurements using the digital substraction angiography (DSA) system implemented software was used to determine the diameter of the target vessel for appropriate sizing of the device.

### Management of Antiplatelet Therapy

**Fig. 1:**
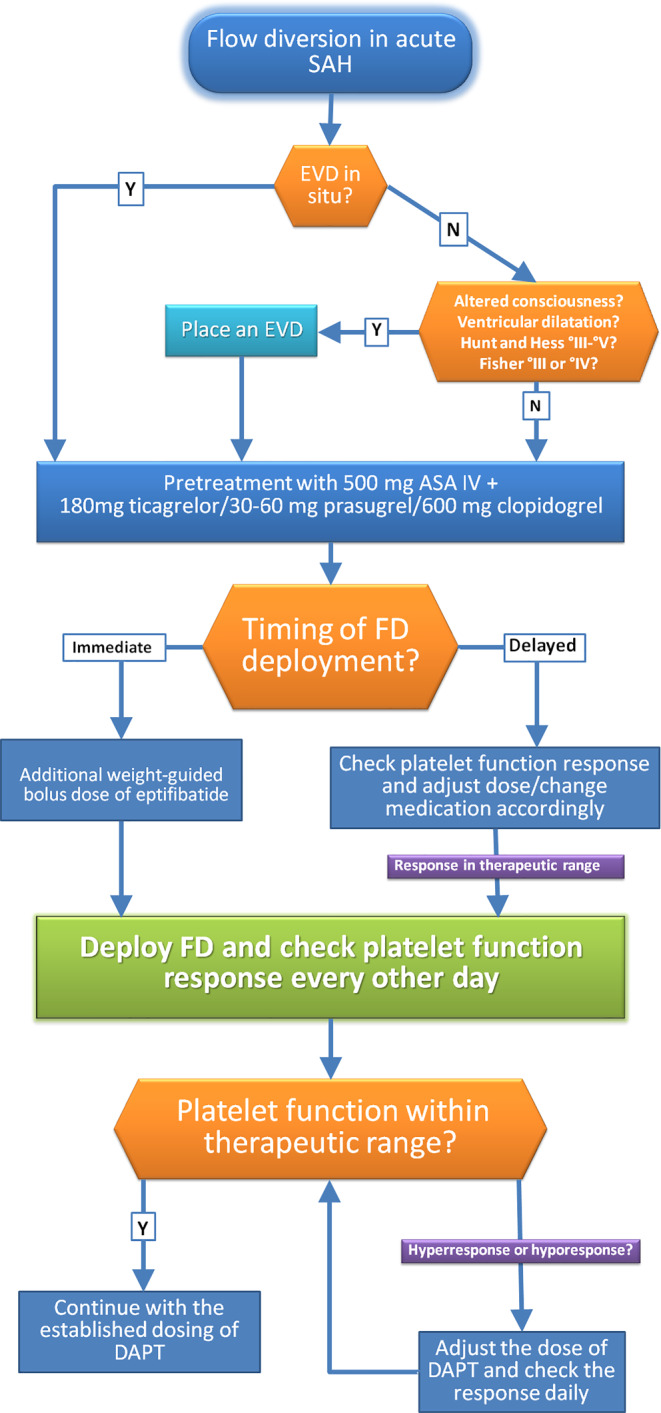
Flow chart illustrating the management of antiplatelet medications when flow diversion is performed in the acute phase of subarachnoid hemorrhage. *SAH* subarachnoid hemorrhage, *EVD* external ventricular drain, *FD* flow diverter, *DAPT* dual antiplatelet therapy, *ASA* acetylsalicylic acid, *Y* yes, *N* no, *IV* intravenous

An external ventricular drain was inserted in all cases, in which the use of a stent or FDS was anticipated. Depending on the timing of the procedure, a loading dose of aspirin (500 mg) and either clopidogrel (600 mg), ticagrelor (180 mg), or prasugrel (30–60 mg) was administered 3 h prior to the procedure and adequate platelet response was tested using the VerifyNow (Accumetrics, San Diego, CA, USA) and Multiplate analyzer (Roche Diagnostic, Mannheim, Germany). In cases with no premedication with DAPT before the procedure, a body weight-adapted bolus dose of intravenous eptifibatide (Integrilin, GlaxoSmithKline, Dublin, Irland) was given in addition to the loading doses mentioned above. The maintenance doses of DAPT were guided by repeated testing of the effectiveness (Fig. 1). In the case of invasive procedures requiring discontinuation of the DAPT, weight-adapted maintenance dose of intravenous eptifibatide infusion was used for bridging up to 2 h before the intended procedure. In the post-acute phase standard daily doses of 1 × 75 mg clopidogrel, 2 × 90 mg ticagrelor or 1 × 10 mg prasugrel for 1 year and 100 mg aspirin indefinitely were maintained.

### Clinical and Radiological Follow-up

As per the institutional protocol, follow-up catheter angiography combined with clinical evaluation are performed 3 and 12 months after flow diversion. Deviations from the standard protocol and extended follow-ups are decided on an individual basis. Assessment of the aneurysmal occlusion after flow diversion is recorded in a simplified fashion as either complete occlusion, neck remnant filling or persistent aneurysm perfusion. Cases of in-stent stenosis without clinical manifestations are managed conservatively as they are usually self-limiting and regress spontaneously. The clinical outcome is assessed on each follow-up by a neurologist or a certified stroke nurse and recorded using the modified Rankin scale (mRS).

## Results

A total of 45 patients (48.9% females) were included who were treated within 30 days from an acute, spontaneous SAH using one or more FDS. The decision to use an FDS was considered as a part of the primary treatment strategy in 41 (91.1%) of the included cases. The decision for flow diversion was secondary after failed surgical attempt in one case (2.2%) and as a second procedure in the acute phase after insufficient primary coiling in three patients (6.7%). The mean age at presentation was 58.8 ± 12.4 years (range 30–80 years). The clinical condition on presentation was good (Hunt and Hess, HH I–II), moderate (HH III) and poor (HH IV–V) in 21 (46.7%), 13 (28.9%), and 11 (24.4%) patients, respectively. The ruptured aneurysms were saccular, blister-like, fusiform and dissecting in 18 (40%), 5 (11.1%), 7 (15.6%), and 15 (33.3%) cases, respectively. Of the aneurysms 22 (48.9%) were in the posterior circulation (45% of which were at the basilar artery) and of these 18 (81.8%) were non-saccular. The mean size of the saccular aneurysms was 6.1 ± 5.3 mm (2–25 mm), all with a dome to neck ratio <1.5. An external ventricle drainage (EVD) was inserted in 19 (42.2%) and 4 (8.9%) cases before and after the endovascular treatment, respectively. Minor hemorrhage along the EVD tract was noticed on cross-sectional imaging in 3 (13%) cases. There were, however, no records of major hemorrhagic complications requiring surgical intervention.

### Endovascular Treatment

Flow diversion was performed with a median latency of 4 days (mean 6.1 days, SD 5.9, range 0–22 days) from the hemorrhage. A total of 10 (22.2%) and 32 (71.1%) patients were treated within 1 day and 1 week from the ictus, respectively. A bolus dose of intravenous eptifibatide was administered along with the loading dose of DAPT during the treatment just before deployment of the FDS in 16 (35.6%) cases, while in the remainder the DAPT was started before the endovascular procedure. Adjunctive coiling of the ruptured aneurysm was performed in 10 (22.2%) cases, while in the remainder (77.8%) flow diversion was performed as a stand-alone procedure. On average 1.62 ± 1.4 devices were used per procedure. A single FDS was used in 31 (68.9%) cases, 2 devices in 9 (20%) cases and multiple devices were used in 5 patients (11.1%). The Pipeline Embolization Device (Medtronic) was used in 7 (15.6%, one of which with shield technology) cases and the p64 flow modulation device (phenox) was used in 36 (80%) cases. A combination of the two devices was used in two (4.4%) cases (both with shield technology). Delivery and deployment of the devices were unproblematic in all but 2 cases (95.6%). Atherosclerotic stenosis immediately proximal to a dissecting V4-aneurysm prevented sufficient opening of the p64 device in one case and the use of a coronary stent to angioplasty the stenosis and opposed the FDS to the vessel wall was necessary. In a second case the mechanical detachment of the p64 device was insufficient at the proximal end of the stent. Balloon angioplasty failed to open the stent completely, and co-axial deployment of a second FDS (PED) was necessary.

### Complications

**Fig. 2:**
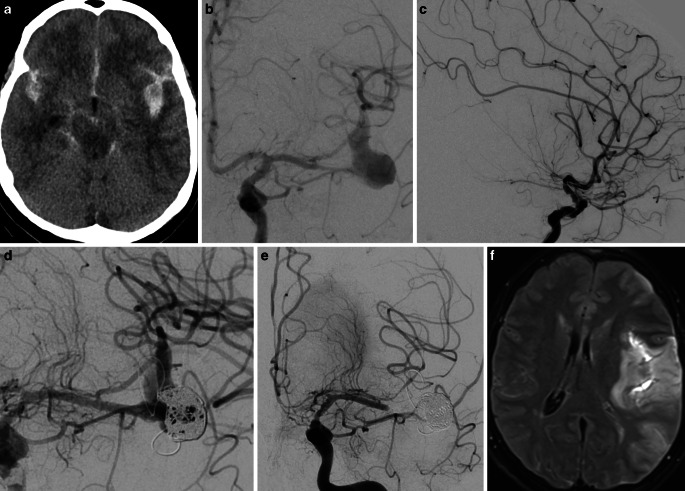
A case of 57-year-old female patient who presented with acute SAH (**a**), Hunt and Hess II due to fusiform, most likely dissecting aneurysm of the inferior trunk of the left MCA (**b**). Temporary occlusion of the parent vessel proximal to the aneurysm demonstrated insufficient leptomeningeal collaterals (**c**). Reconstructive treatment with partial coiling and telescopic deployment of three FDS from proximal to distal was performed (**d**). Stent thrombosis and refractory vasospasm (**e**) resulted in a large MCA infarction (**f**) and eventually death. **a** axial non-contrast computer tomography (NCCT), **b** frontal DSA after left internal carotid artery (ICA) injection, **c** lateral DSA after left ICA injection with inflated balloon in the inferior trunk of the MCA, **d** final run after partial coiling and flow diversion, **e** frontal left ICA injection 9 days after treatment, **f** axial FLAIR (fluid attenuated inversion recovery)

**Fig. 3:**
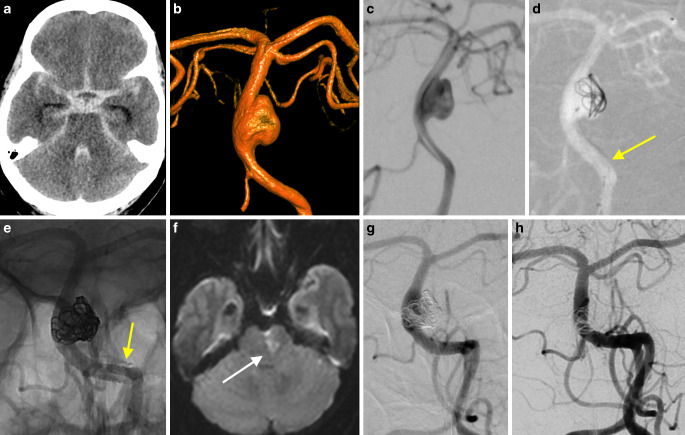
This 62-year-old female patient was admitted with diffuse SAH (**a**), Hunt and Hess grade 3 due to a wide-neck side-wall aneurysm of the basilar artery (**b** and **c**). The aneurysm was treated with partial coiling and flow division using the p64 flow modulation device in jailing technique (**d** and **e**). Right hemiparesis was noted after weaning and a corresponding left mediolateral pontine ischemia was confirmed on MRI (**f**). The emergently performed diagnostic angiography (**g**) showed no stent thrombosis. The most recent follow-up angiography performed 1 year after treatment shows complete exclusion of the aneurysm and patency of the stent (**h**). **a** axial NCCT, **b** 3D-VRT (Volume Rendering Technique), **c** working projection, **d** FDS-assisted partial coiling, the *arrow* points to the yet undetached FDS, **e** unsubstracted view of final run after partial filling of the aneurysm and detachment of the FDS (*arrow*), **f** axial DWI, **g** and **h** frontal views of left vertebral injections

Procedure or device-related complications (including asymptomatic) were recorded in a total of 6 (13.3%) cases and included 3 intraprocedural complications, 2 postprocedural complications in the acute phase and 1 delayed complication. Intraprocedural rebleeding from the dissected right V4 segment occured in one case before catheterization and resulted in massive and fatal raise of the ICP despite the already inserted EVD. In one case, a combination of stent thrombosis and refractory cerebral vasospasm (CVS) resulted in fatal middle cerebral artery (MCA) infarction (Fig. 2). One patient suffered a left paramedian pontine ischemia after the procedure. There was, however, no evidence of stent thrombosis in the immediately performed DSA (Fig. 3). Intraprocedural thrombus formation in or in the vicinity of the device(s) was observed in two cases (4.4%) and both resolved after an intravenous bolus of eptifibatide with no resultant ischemia. In one case, incompliance with the DAPT resulted in asymptomatic stent thrombosis, which was discovered on a routine angiographic follow-up. In total, procedure-related complications resulted in permanent morbidity or mortality in 3 (6.7%) patients.

### Clinical Outcome

There were no records of rebleeding from the target lesion after treatment. In total, 68.9% of all included patients in this series achieved an excellent clinical outcome (mRS 0‑1), 5 patients (11.1%) died in the acute phase, including the two procedure-related mortalities already mentioned. The other mortalities were procedure-unrelated (1 case of fatal pulmonary embolus, 2 cases due to the primary hemorrhage). One patient died due to medical complication 2 months after discharge, and one patient was lost to follow-up. Among the remaining 38 (84.4%) patients, a total of 31 (81.6%) achieved an excellent clinical outcome according to the latest clinical follow-up.

### Radiologic Outcome

Immediate complete occlusion was achieved in 6 (13.3%) cases while reduced filling and unchanged filling of the aneurysm were observed in 29 (64.4%), and 10 (22.2%) cases, respectively. Early mid-term and long-term angiographic follow-up studies were available for 37 (94.9%) and 35 (89.8%) of the surviving 39 patients, respectively. Total or near-total occlusion was observed in all patients with available angiographic follow-ups. Complete occlusion of the aneurysm or complete reconstruction of the dissection was achieved in 34 (94.6%) patients. In two cases a minor neck remnant was observed on the first follow-up after 6 months, but both did not present to the scheduled second follow-up. In one case with a dissecting right V4 aneurysm a minor endoleak around the struts of the FDS persisted on follow-ups. There were no records of a recurrence of the treated lesion.

## Discussion

### Safety and Efficacy

Managing intracranial aneurysms by mean of endoluminal flow diversion offers some significant advantages over endosaccular treatment strategies, especially when dealing with ruptured aneurysms. These include but are not limited to avoiding wire and/or catheter manipulation inside the aneurysmal sac and durability of aneurysmal occlusion. The potentials of FDS for treatment of otherwise challenging ruptured aneurysms was recognized early on [9]. The acute phase of subarachnoid hemorrhage, however, poses several challenges to the use of FDS and experience of their use in the acute phase is thus still limited. The results thus suggest an excellent efficacy and a good safety profile of FDS for the management of acutely ruptured aneurysms as an adjunctive device or stand-alone treatment. In this series, total or subtotal occlusion was achieved in all patients with follow-up studies with no records of rebleeding between the treatment and the angiographic cure of the target lesion. The rate of treatment-related permanent morbidities was 6.7%, which compares well with the conventional endovascular strategies [10]. The favorable complication rate in this series could be explained by the increased use of FDS in the acute setting and including more “less complex” aneurysmal morphologies as more experience was gained with this novel strategy over the years. Almost 75% of the included cases were performed in the second half of the study period. A recent meta-analysis of 20 studies including 233 patients treated with FDS for acutely ruptured aneurysms reported an almost 90% rate of total or subtotal occlusion at a mean of 9.6 months and although the immediate occlusion rate was only 32%, the rebleeding rate was nonetheless low at 4% suggesting that aneurysmal re-rupture is not a significant concern with the use of FDS despite the persistent filling [8]. The overall complication rate was 18% with 7% treatment-related morbidities and comparable rates of hemorrhagic and thromboembolic complications. When comparing the complication rate associated with the use of FDS against other endovascular or surgical modalities for treatment of ruptured aneurysms, it is essential to keep in mind that FDS is usually considered for the treatment of challenging aneurysms, where other modalities are deemed too risky or unfeasible [7]. Giant aneurysms and those located at the posterior circulation are associated with higher complication rates after treatment with FDS [11]. The more durable results might mitigate the high complication rates after flow diversion for ruptured aneurysms and thus decreased rates of late rebleeding in comparison to conventional endovascular coiling where high recurrence rates of ruptured aneurysms is a significant drawback [5, 12]. Long-term follow-up studies suggest high rates of progressive and durable occlusion after flow diversion for unruptured aneurysms, but the evidence is still limited [3, 13, 14]. The optimal timing of flow diversion in the acute phase is not established. Deployment of the procedure would theoretically risk an early re-rupture before treatment but would allow more optimal stabilization of the clinical condition and more adequate premedication with antiplatelet medications. Approximately half of the patients in the study were treated within 72 h from the ictus, and the treatment-related complications were encountered in those treated within 48 h. A meta-analysis comparing the complication rate associated with early (less than 2 days) vs. delayed (mean latency of 4.2 days) flow diversion in the setting of acutely ruptured aneurysms reported no overall statistically significant difference [15]. Early treatment for blister or dissecting/fusiform aneurysms was associated with a low complication rate in comparison to saccular aneurysms. First coiling in the acute phase followed by staged flow diversion for ruptured giant aneurysm appears to be safe and effective [16].

### Management of Antiplatelet Therapy

A major concern with the use of FDS is the need for DAPT. Patients with high-grade SAH frequently need invasive procedures, such as CSF diversion, tracheostomy, gastrostomy, etc. The need for effective DAPT would thus theoretically increase the rate of hemorrhagic complication of such procedures. With the appropriate precaution, however, the frequency of hemorrhagic complication under DAPT can be minimized. There were no records of major hemorrhagic complications related to the use of DAPT in this series, and although 51.1% of the patients received an EVD, minor hemorrhagic manifestations were observed in only 13% of these cases and were without clinical sequelae. A recent single center series of 47 cases of stenting in the acute phase of aSAH (including 20 FDS) reported no hemorrhagic complications of the reviewed cases [17]. The favorable safely outcome with respect to the hemorrhagic complication was attributed by the authors to the low threshold for EVD insertion before DAPT in cases where the use of a stent was anticipated.

Interestingly, despite adequate response to the DAPT in platelet reactivity testing, the authors reported an intraprocedural rate of thromboembolic complications of 9%, all of which occurred in patients who underwent stent-assisted coiling and there were no thromboembolic complications in patients who underwent flow diversion. A review of the affected cases revealed a platelet response at the higher end of the accepted spectrum by the protocol used and led to change into a tighter protocol. Indeed, achieving sufficient antiplatelet response for safe use of endoluminal devices in acute SAH might be challenging. Besides the increased thromboxane release and an associated increase in platelet activation and aggregation, patients with acute SAH might suffer decreased gastrointestinal motility and absorption which might render standard dosing of antiplatelet medication insufficient and individualized dose adjustment might be necessary [18–21]. Interestingly, a meta-analysis of stent-assisted endovascular treatment of ruptured aneurysms showed higher rates of thromboembolic complications compared to hemorrhagic complications. Postprocedural administration of DAPT was significantly associated with higher thromboembolic complications (in comparison to preprocedural and intraprocedural administration) [22]. As a certain proportion of patients might not respond to treatment with clopidogrel, ticagrelor and prasugrel might be more suitable for quicker and more efficient inhibition of ADP binding to platelet P2Y12 receptors when considering the use of FDS [23, 24]. Although the recommended loading dose of prasugrel is 60 mg, half loading was sufficient for effective inhibition in over 98% of patients in a retrospective series of 138 patients (including 12 ruptured aneurysms) treated with FDS [25]. Cangrelor is the only approved intravenous P2Y12 inhibitor. Its rapid onset of action and short half-life make it an attractive alternative to the oral thienopyridine agents in the acute phase of SAH, but the clinical experience is still minimal, and the high price is a significant disadvantage [26]. A promising recent advancement has been the surface modification to reduce the inherent thrombogenicity of FDS [27–29]. Manning et al. used the PED with shield technology under single antiplatelet therapy in treating 14 patients with ruptured intracranial aneurysms and reported no hemorrhagic or thromboembolic complications in the subgroup that did not receive postinterventional heparin infusion [29]. Until establishing the efficacy and safety of such coatings in a large clinical trial, the use of FDS will remain limited by the need for DAPT.

### Limitations

The major limitation of the presented study is the single center, retrospective, and single arm design. The use of only two of the available FDS might limit the generalizability of the results to other commercially available FDS. The evaluation of clinical outcome is confounded by the complex nature of the disease and difficulties to assess the real impact of device-related complications on the final clinical outcome. Subgroup analysis of procedural safety and clinical outcome according to the different types of treated aneurysms or implemented devices/techniques categories is limited by the cohort size.

## Conclusion

In this series of 45 patients treated in the acute phase of spontaneous SAH, the use of FDS achieved a very high rate of complete angiographic occlusion on mid-term and long-term follow-ups with excellent safety profile. Despite the low rate of immediate complete occlusion, no rebleeding occurred during the latency period. Appropriate timing and adequate management of DAPT can reduce the rate of both hemorrhagic and thromboembolic complications. Although plain coiling is and probably will remain the mainstay treatment for anatomically suitable aneurysms, the continuous technical improvements and growing clinical experience with flow diversion might lead to more frequent use of this technique in the management of acutely ruptured aneurysms.
